# Planning Individual and Population-Based Interventions in Global Health: Applying the DEA-A Framework to Promote Behavioral, Emotional, and/or Cognitive Change among Stakeholders

**DOI:** 10.3390/ijerph21030378

**Published:** 2024-03-21

**Authors:** Guillaume Broc, Jean Baptiste Fassier, Stéphane Raffard, Olivier Lareyre

**Affiliations:** 1 EPSYLON EA 4556, Paul Valéry Montpellier 3, University of Montpellier, 34090 Montpellier, France; stephane.raffard@univ-montp3.fr (S.R.); olivier.lareyre@univ-montp3.fr (O.L.); 2 UMRESTTE UMR T 9405, Université Lyon, Université Claude Bernard Lyon 1, 69002 Lyon, France; jean-baptiste.fassier@univ-lyon1.fr; 3 Occupational Health and Medicine Department, Hospices Civils de Lyon, 69002 Lyon, France; 4 University Department of Adult Psychiatry, CHU Montpellier, 34090 Montpellier, France

**Keywords:** DEA-A framework, global health, behavior change, cognitive change, emotional change, intervention mapping, program planning, chronic disease

## Abstract

Addressing health challenges that impact human well-being requires a comprehensive, interdisciplinary approach that would be at the crossroad of population-based prevention and individual-level clinical care, which is in line with a Global Health perspective. In the absence of a unifying theoretical framework to guide such interventions, a Dynamic Ecosystem Adaptation through the Allostasis (DEA-A) framework has been proposed, emphasizing the functional adaptation of individuals and organizations in symbiosis with their living ecosystem. While a conceptual model has been presented, this methodological contribution aims at illustrating the practical application of the DEA-A framework for planning Global Health interventions. The methodology combines Intervention Mapping and Cognitive and Behavioral Theory, extended to the ecosystem. Practical guidelines and supporting tools are provided to help public health providers and clinicians in establishing a functional ecosystem diagnosis of the issue; defining not only behavioral, but also emotional and cognitive change objectives (allostasis targets) expected for each stakeholder; and designing intervention plans targeting determinants of these allostasis. The discussion addresses implementation and evaluation perspectives of interventions based on the DEA-A framework, emphasizing the importance of considering change in its processual and ecosystem complexity. Lastly, encouragements for a deeper understanding of individual and ecosystem homeostasis/allostasis processes are made in order to promote more functional interventions.

## 1. Background

The relationship between human health and ecosystem health has never been more evident than in recent years. Recurrence of global crises encompassing health, environmental, economic, political, and social aspects has contributed to deteriorating health and well-being, the destabilization of institutions, and an increase in social inequalities regarding exposure to risks and healthcare access [1]. As a result, the definition of health has evolved in order to better address these concerns about human adaptation within a constantly changing ecosystem. A consideration of the ecosystem echoes current health acceptances underpinning the Global Health approach. The World Health Organization defines Global Health as ‘an area for study, research, and practice that places a priority on improving health and achieving equity in health for all people worldwide’ [2], suggesting a comprehensive, interdisciplinary approach to health that involves the integration of various disciplines and the ambition to synthesize population-based prevention with individual clinical treatment.

Such a perspective requires that health interventions take into account the multidimensionality of the problem, which involves determining factors at the internal level of the actors, dynamic relationships between the stakeholders, as well as contingencies in the real-world (political, social) environment [3,4]. The use of a theory through an evidence-based program planning method is widely recommended [5,6,7]. Theory serves as a framework for guiding intervention strategies, ensuring that complexity is addressed at every stage of the planning process. Beyond the intervention’s content alone, theoretical anchoring allows for a deeper understanding of contextual factors, processes, and mechanisms of the intervention, as well as the applicability and transferability factors that need to be identified for proper operationalization and evaluation [8,9,10]. In terms of expected benefits, it has been demonstrated that theory-based interventions can be more effective in supporting health-related changes when they are adequately mapped [11,12,13,14,15]. Logic models have been proposed to define program theories in the ‘real-world’ [3,16]; however, to our knowledge, there is no model integrating explanatory theories of human functioning that would enable us to further investigate the interplay in the adaptation process of stakeholders and ecosystems, as well as the impact of these adaptations within the context of intervention. Such an integrative framework would support Global Health objectives by guiding the planning of interventions that integrate individual-based (IBi) clinical approaches and population-based (PBi) prevention approaches.

To guide such interventions, a Dynamic Ecosystem Adaptation through an Allostasis (DEA-A) framework has been proposed [17]. It articulates the contributions of models in the psychology of adaptation, particularly the Homeostasis/Allostasis theories [18,19,20,21,22], as well as an ecosystemic perspective of human development [23,24,25], emphasizing the process of adaptation for both individuals and organizations within their living ecosystem. According to the DEA-A framework, adaptation occurs on two levels. The first considers the intra-system dynamics of actors or organizations (A/O). Homeostasis–Allostasis governs this process, in which stress (arousals) is induced when the functioning of the A/O is challenged, and, in response, behavioral, emotional, and/or cognitive regulations occur (allostatic regulation). Feedback from this adaptation is viewed in terms of recovering homeostasis and the inherent costs incurred (allostatic load). On the second level, ecosystem dynamics are considered, which integrate the mutual influences that contribute to the adaptation of A/Os within an environment. There are a number of nested systems involved in this dynamic: the ontosystem (i.e., intra-system level described above), the microsystem (relationships with other A/Os), the mesosystem (relationships between A/Os), the exosystem (political and institutional environments), the macrosystem (societal and cultural environments), and the chronosystem (temporality, transient nature).

The DEA-A framework aims to facilitate the establishment of a functional analysis, providing insights into the reasons, processes, and consequences of how each A/O adapts and influences the prevailing situation. A diagnosis in this regard could lead to recommendations for fostering more effective adaptations from stakeholders in symbiosis with their living ecosystems. In more precise terms, it involves promoting and supporting A/Os in transitioning from a functioning Profile A (characterized by a set of cognitive, emotional, behavioral regulations) to a functioning Profile B that is beneficial to both them and their ecosystem in the context. While the DEA-A framework has been conceptually presented (see Broc, Brunel and Lareyre, [17]), guidelines for its practical application have yet to be provided to ensure proper theoretical anchoring of interventions in a way that is consistent with the Global Health approach.

## 2. Objective

The objective of this methodological contribution is to present a procedure for planning Global Health interventions while using the DEA-A framework, along with the supporting tools than can be applied in IBi and PBi contexts in light of such an epistemology.

## 3. Suggested Method

The Intervention Mapping [IM] protocol [5,26,27] is one of nearly forty methods dedicated to the Planning of Health Promotion Programs (see Carbonnel and Ninot [28] for a review). While these other guidelines can be followed, we believe that IM remains fundamentally close to a DEA-A framework, allowing its application to program planning to be structured more efficiently. A major benefit of IM is its ability to integrate theory and evidence within an ecosystem-based approach through participatory research. As part of the planning process, scientific evidence and the experience of patients and professionals are triangulated to co-develop, implement, and evaluate an intervention with the stakeholders’ community. The IM process is described in six steps, as follows: (1) logic model of the problem–strategic committee; (2) program objectives: logic model of change; (3) program design; (4) program production; (5) implementation Plan, and (6) evaluation plan. As part of this contribution, only the first three steps leading to the design of the intervention will be addressed, while describing for each the utility and operationalization of a DEA-A framework. Health providers will be provided with illustrations for both PBi and IBi as Supplementary Material to facilitate their planning of interventions in both contexts (see File S1) (Note: *in the IBi context, healthcare providers are considered to be any actor capable of coordinating individual support for the person. This role can be fulfilled in clinical practice by the patient’s psychologist, the primary care physician, a primary care provider, the medical advisor, a patient-partner, or even the patient themselves in certain specific circumstances (particularly in the absence of a defined diagnostic or care pathway). This service can also be provided by health insurance mutuals. In the PBi context, the healthcare provider refers to any actor who may promote the program, whether they operate (non-exhaustively) on behalf of a research laboratory, a public health agency, a political entity, a company, an association, or any other organization or structure. New professions such as “Global Health consultants” can also be envisaged in this context*.).

### 3.1. Step 1. Logic Model of the Problem–Strategic Committee

The objective of this step is to make a descriptive examination of the situation in all its complexities (i.e., key issues, determinants of the problem, characteristics of the environment, actors involved, etc.). An integrated reading combining scientific and experiential information is conducted by the promoter in collaboration with a committee of stakeholders. A charter that defines the values, needs, and commitments of the group can be used to formalize collaboration with this committee. Clinically, the first step would involve the analysis of both the request and the patient’s situation, with the prior establishment of a therapeutic alliance which permits collaboration. It is possible for clinicians in liberal or institutional practice to collaborate with other actors throughout the integrative care process, as is the case with dyadic care (e.g., marital, family, systemic therapies) or protocol-referring consultation groups (e.g., multidisciplinary team meetings/medo-social MDTs, hospital day care, expert centers).

*DEA-A Framework’s Contribution (see Boxes S1 and S4 in File S1)*

**Figure 1:**
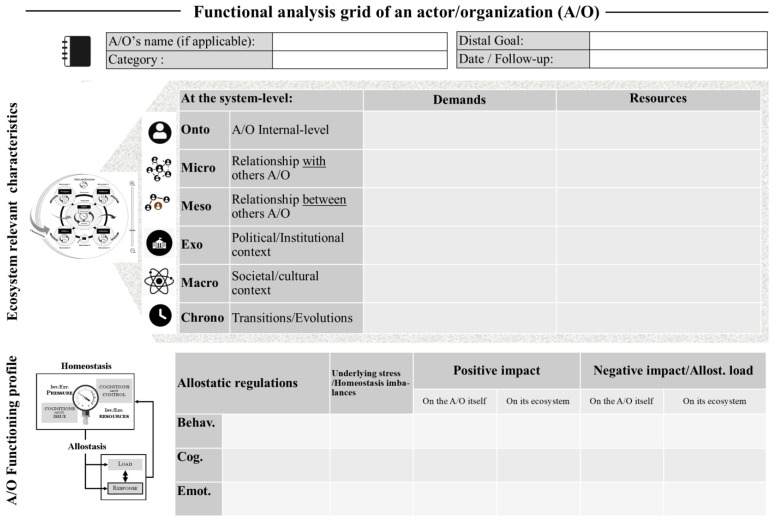
Step 1 Functional analysis grid of adaptation according to the DEA-A framework.

As such, the DEA-A framework enables a functional diagnosis or audit of the situation by describing an A/O’s allostatic (emotional, cognitive and behavioral) responses, identifying the imbalances underlying these regulations and the nature of the stress, analyzing the conditions contributing to demands or resources within each stratum of its ecosystem (from the ontosystem to the macrosystem), and verifying whether this allostasis is functional for both the A/O and its ecosystem as a whole. As shown in Figure 1, a functional analysis grid can be used for this purpose. The DEA-A grid is based on functional analysis worksheets that are used in Cognitive Behavioral Therapy (CBT) to examine the causes and consequences of behavior in context [29], but it is adapted here for ecosystem diagnosis.

The analysis grid should be completed for each category of A/O (for PBi) or each key A/O (for IBi). The empty fields at the top provide information about the A/O (and, where applicable, its microsystem of belonging) as well as the date or, where appropriate, the date of follow-up in order to facilitate a dynamic and procedural evaluation of the A/O’s situation over time. In the first main section, the functioning of the ecosystem and its influence on the A/O are questioned. This ontosystem is intended to document, if possible, elements from the A/O’s internal environment that are important to its homeostasis (e.g., chronic illnesses that affect organs and their functions). The second section discusses the functioning of the A/O (in terms of behavioral, cognitive, and emotional regulation) as well as its impact on the ecosystem. As it stands, self-assessments (either through interviews, scales, or questionnaires) only provide access to information which has been elaborated (e.g., conscientized) or reconstructed by the individual through cognitive processes, and thus integration with other data collection methods, such as hetero-assessment (e.g., directed interviews or observation), objective measures (e.g., biomarkers), or even implicit measures, is required. The health care provider can either rely on indirect sources (e.g., information from another actor or cross-checking information from literature reviews and/or documentary research) or leave the item missing when the functioning of an actor cannot be explored directly. In both cases, the validity of the evaluation should be considered. The completed grids will allow the DEA-A framework presented in the conceptual article to be informed, thereby providing a logical model of the situation. An editable DEA-A grid can be found in File S2.

### 3.2. Step 2. Program Objectives: Logic Model of Change

The purpose of Step 2 is to identify the program change objectives that will be further targeted by the intervention components. Step 2’s deliverables are matrices of change in which the targeted performance objectives for each actor are listed in rows along with their determinants in columns. In IBi, this process would include breaking the clinical demand down into specific or intermediate objectives to be achieved through therapy.

*DEA-A Framework’s Contribution (see Boxes S2 and S5 in File S1)*

An ecosystem’s physiognomy can be affected both by action and inaction. The intervention objectives must therefore include not only behavioral, but also cognitive and/or emotional changes. It should also be noted that behavior change is not always necessary or desirable (Note: *there is, sometimes, no behavioral solution, or at least no ideal behavior to promote. Furthermore, it may be unwise to instruct adults on how to conduct themselves, especially as there is a nondescript range of options. Rather, it may be appropriate to let A/Os room to maneuver their own actions/decisions and to rely solely on fostering a cognitive and emotional functioning which provides the conditions necessary for these behavioral regulations, in whatever form, to naturally emerge. The individual who is more attentive to both his needs (ER) and those of others (CR) will behave more coherently (BR) through his participation in the well-being of his ecosystem without being prescribed any particular behavior to adopt. This approach will prevent health providers from multiplying actions by as many interventions, as there are health behaviors that should be promoted*). Primarily focused on helping behaviors to be promoted, the change matrices now consider the entire spectrum of behavioral regulations [BR] by also integrating problem behaviors to inhibit, as well as considering other forms of cognitive [CR] and emotional [ER] regulation as objectives of change. In other words, it involves developing a behavioral, cognitive, and/or emotional functioning profile for each A/O that will be achieved through the intervention. In this way, further adaptations could be made spontaneously by the A/O itself that would be perfectly aligned with their needs and in symbiosis with the ecosystem as a whole. When these targeted regulations have been identified and prioritized, they will formalize the ‘change objectives’ lines in a given A/O matrix (see Table 1). Following the DEA-A framework, both the expected benefit and the associated allostatic load should be considered for the A/O as well as its ecosystem. In addition, as adaptation is a process, objectives can be arranged according to a logical sequence (even to the extent that a given target regulation determines a second one), and should therefore be prioritized in the program sequence (such as in exposure therapies, where behavior determines subsequent cognitive functioning).

**Figure 2:**
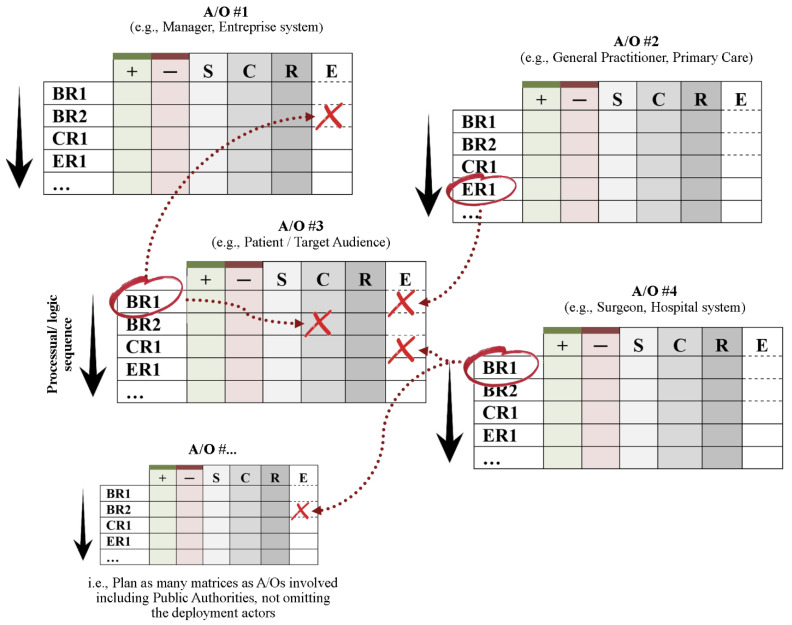
Articulation between matrices providing a preliminary overview of the ecosystem process of change. BR = Behavioral Regulation; CR = Cognitive Regulation; ER = Emotional Regulation; S = Stress; C = Cognitions; R = Resources; E = Ecosystem. A/O = Actor or Organization. The cross means that the allostasis of an A/O determines its own future regulations and those of other A/Os.

Therefore, it remains to determine in columns the factors that influence the behavioral, cognitive and/or emotional functioning of the A/O; again, the DEA-A framework can be of assistance. Because A/O is driven by the search for stability/plasticity, any change must be based on bringing the system into homeostatic imbalance and resolving stress that is incompatible with these changes. To obtain targeted regulation, the determinants ‘S’ for ‘Stress’ should be identified in order to determine the nature of the arousals to be induced or reinforced, and those to be tempered. The allostatic loads expected for targeted regulations may be included in these calculations. Other determinants concern cognitions about issues and about resources that influence regulation probability in the DEA-A framework, which are grouped here into the category ‘C’ as ‘Cognitions.’ The factual internal resources of the A/O are called ‘R’, which provide the means for initiating, maintaining, or restricting given regulations. As the DEA-A framework is cyclical, resources (such as health) must be considered both as determinants and outcomes. It is therefore possible to modify them to support other regulations, including those targeted by the intervention. The function of external resources is the same, but they are dependent upon the context, notably other A/Os’ regulations. The determinants are designated ‘E’ for ‘Ecosystem’. Health providers will therefore consider in columns the key SCR-E determinants of each regulation objective derived from the DEA-A framework. In the ecosystem, A/Os are interrelated, so matrices must be considered as interconnected. This means that any regulation (BR, CR, or ER) sought through an A/O may result in the configuration of a determinant ‘E’ in the DEA-A matrix of another A/O, contributing to its own change process (see Figure 2). In File S1, Boxes S2 and S5 illustrate how references to other cells within an A/O’s matrix, as well as references to the matrices of other A/O’s, help consider the change in its temporal and ecosystem dynamics over time. The file that contains all the matrices depicts the logical model of change. It is expected that this model will be limited to the patient matrix in the IBi context, or even integrate the dyad/caregiver matrix in case of systemic therapy (e.g., marital/family). File S3 contains an editable DEA-A matrix with different spreadsheets (one for each A/O).

### 3.3. Step 3. Program Design

The purpose of this step is to specify the content of the intervention, i.e., the components which are expected to produce the effects on the objectives of change through the mechanism(s) of action postulated (here, impacting the column determinants of the matrix). Program active principles are derived from theory and evidence-based change methods, as well as from more experiential strategies (such as those used by patients or professionals on a daily basis). The deliverable of Step 3 is a preliminary version of the intervention plan (i.e., logic model of the program) which describes the program strategy and its components.

*DEA-A Framework’s Contribution (see Boxes S3 and S6 File S1)*

The literature contains an abundance of determinants that overlap significantly. In designing an intervention, it would be more operational to reduce these determinants to the sole SCR-E categories. According to the DEA-A model, one PBi scope of action specifically targets individuals within the ecosystem (e.g., general practitioners, managers, caregivers). Like IBi, PBi could benefit from a variety of clinical psychotherapy techniques, including Cognitive Behavioral Therapy (CBT) [30], Acceptance–Commitment Therapy ACT [31] or Schema Therapy [32]. Several of these techniques, as well as many others, have been outlined in the Taxonomy of Behavior Change Techniques [BCT] suggested by Michie et al. [33,34]. A non-exhaustive list of intervention techniques useful in targeting SCR-E determinants is presented in Table 2.

For various reasons, this table is only indicative. First, not all techniques are listed, in particular those that can be proposed by the A/Os themselves (through the interview and, where appropriate, stakeholder committee) and/or left to their own initiative, ensuring their relevance/validity in the context. Secondly, due to the porosity of the perimeters of effect of each technique, exacerbated by the heterogeneity of their operationalization, the breathing exercises (relaxation) were placed in the ‘S’ column as they are considered a component of symptom relief, whereas they may actually increase self-regulation skills and therefore the individual’s ‘R’ resources. As there is no ideal classification that can be achieved, this indicative proposal only provides ideas to professionals and must be interpreted in light of the specific circumstances. Ultimately, it is up to the health care providers to determine which techniques to use and the sequence in which they should be administered into the intervention plan. Among existing techniques, they may select the most appropriate ones for addressing several SCR-E determinants and/or several change objectives at the same time. It may also be beneficial to take concerted actions to address the needs of several stakeholders (e.g., coordination of care via an instant messaging app with the patient).

## 4. Discussion

We propose both a procedure and supporting tools for planning IBi or PBi in Global Health settings based on the DEA-A framework in this methodological contribution. In this regard, a strategy based on the IM protocol was recommended. The IM protocol lends itself particularly well to the application of a DEA-A framework for the planning of complex interventions, taking into account ecosystem dynamics. We have demonstrated here the three preliminary steps of IM leading to the formalization of the program objectives and the design of the intervention.

An important feature of the DEA-A approach in this view is to systematically consider the living ecosystem surrounding the A/O. A systematic search for symbiosis should therefore be conducted, at a minimum, through indirect knowledge of the other systems involved. Another characteristic of the DEA-A framework is to promote optimal behavioral, cognitive, and emotional functioning rather than focusing solely on behavior change. Thus, the action would become cost-effective by expanding its effects to other contexts dependent on such functioning. Moreover, as emphasized in the Homeostasis Theory of Well-Being [18,19], the cyclical nature of the Homeostasis–Allostasis process implies that determinants and outcomes are interchangeable statuses, indicating that behavior may affect subsequent cognitive, emotional, and behavioral regulation. This circularity has raised the question of explicitly identifying behavior as a determinant in the DEA-A matrices (i.e., along with the SCR-E determinants). To facilitate the planning process, the decision was made to include behavior either as a component of intervention (e.g., in exposure therapies, where it determines cognition) or as an objective change BR. Even so, the reference system in the cells of the matrices enabled establishing the place of this behavior in the process of change (i.e., sometimes as a BR determined by SCR-E, sometimes as one of the determinants of a subsequent regulation).

The purpose of the DEA-A framework is to serve as a guide, not a shackle, when designing programs. In this regard, it is vital to maintain the health provider’s room for maneuvering. The flexibility is found in the determination of intervention components, giving more latitude to stakeholders regardless of whether they are the target of the intervention or those in charge of its deployment [4]. It is indeed illusory to expect control or even to predict all the specifics of a situation. Even the most predictive model may contain some residual error that is best addressed indirectly by relying on the actors (patients and healthcare staff) who are much more likely to accommodate these singularities. In other words, intervention must be defined by its function, not its form [35].

Furthermore, it is necessary to consider the intervention environment through the conditions of exposure and the context of delivery [3,9]. The appropriation and deployment factors are questioned in Step 4 (Program Production) and Step 5 (Implementation Plan) of Intervention Mapping [5]. It is important to note that the DEA-A framework can still be useful during these phases in regard to optimizing the reception, handling, and observance of the intervention, namely by taking into account the stakeholders’ cognitive and emotional resources and their allostatic load, as well as to optimizing the implementation of the intervention, such as providing DEA-A matrixes to all actors responsible for the implementation (e.g., by expecting from them to provide this information and adopt this posture when delivering the tools).

Last but not least, considering the need to evaluate future programs based on the DEA-A framework (see IM Step 6: Evaluation Plan), methods that take into consideration the situation and the contribution of the “real-world” context of intervention are to be preferred [4,36,37]. Ecological Momentary Assessment, for example, can be used to assess an individual’s daily experiences, behaviors and emotional reactions [38,39]. Various mixed designs, including the Single Case Design, may be indicated as well, in which quantitative measurements are combined with qualitative or clinical contextualization [40]. There is no doubt that longitudinal studies are required to capture the ecosystem process of adaptation. Machine learning or AI can therefore be incorporated into both PBi and IBi evaluations of interventions [9,41,42,43]. The same applies to Network Analyses that examine dynamic relationships among stakeholders over time [44,45,46].

For the deployment of such a methodology in practice, we identified potential actors who could serve as Global Health providers [GHPs]. As a matter of fact, the scope and competence of these stakeholders will remain evidently limited. Therefore, healthcare providers need to collaborate with partners, including users, to ensure that they cover all areas of expertise in a given situation. More than simply referring them to the professional, the DEA-A approach involves the GHP coordinating monitoring and fostering real collaboration with all stakeholders. We believe that this undertaking, albeit complex, is essential in the practice of Global Health. It is worth noting in this regard that the DEA-A method draws heavily from the Intervention Mapping protocol (steps, change matrices by stakeholder, etc.), the effectiveness of which has been demonstrated in clinical and preventive contexts [47,48,49].

Such an approach is found in population-based interventions in which the promoter acts on an organizational, political, and societal level and can make use of the DEA-A framework to further integrate the adaptation processes of systems and actors during intervention planning. In health prevention, this GHP, for example, is a mission officer (e.g., promoting healthy diet and physical activity among adolescents) or a coordinating physician (for organized cancer screening). They undertake the analysis of the functionality of the adaptation of the target audience (e.g., adolescents), but also of the stakeholders in the situation, preferably on a territorial scale (e.g., school personnel, nurses, cafeteria staff, directors, families, local farmers, the municipality, etc.). This analysis (Steps 1 and 2), followed by the intervention (Step 3), is conducted at an organizational level (e.g., directives, constraints, and resources allocated to the school cafeteria), but also at the individual level of each actor (e.g., the cafeteria worker, not only from the perspective of their status/role in the institution, but also from their personal functioning, i.e., cognitions and emotions related to the issue). Functional analysis grids and matrices are carried out as needed for each actor, category of actor, and organization, and they support the planning (see File S1). At each step, at least one representative from each category of stakeholders participates in the planning, which already constitutes an intervention in itself [50].

In the clinical contexts, certain devices lend themselves well to the application of the DEA-A method, such as hospital day care, specifically in expert centers, or even multidisciplinary team meetings. A private clinician may register with a healthcare network in the territory in which they practice. The process is facilitated by an exercise in an institution or within a professional association (e.g., medical office, specialist health center, multi-professional health center, etc.). For coordination of DEA-A approaches, they can use tools developed for caregivers, such as instant messaging applications (rapid information exchange between caregivers, transmission of documents, shared agendas to plan MDTs involving one or more patient records), which may also be applicable to other actors in the field (patient associations, community representatives, public policies, etc.). In the region, multidisciplinary intervision programs may also be developed. IBi and PBi practices should eventually evolve towards a comprehensive Global Health approach, which will require future GHPs to be trained, not to master all areas of expertise, but with the methodology enabling them to bring together all the experts on the case (i.e., training in group facilitation, in piloting participatory projects, and in mastering the methods and tools that this contribution presents, etc.).

Efforts have been made in this contribution to describe the transversal application of a DEA-A framework in both PBi and IBi contexts in line with a Global Health perspective. Still, further contributions will be necessary to specifically deepen these explanations and experiment either in population approaches or in a clinical setting. The article also remains limited in its ambition to change the practices of healthcare providers. It will not replace the necessary training and supervised practice of future GHPs, which alone ensure mastery of any method.

## 5. Conclusions

This contribution addresses epistemological and methodological concerns surrounding the new challenges underpinned by the Global Health approach by developing guidelines and supporting tools based on a Dynamic Ecosystem Adaptation through the Allostasis (DEA-A) framework. It is reasonable to assume that the contribution could awaken health providers to the importance of reconciling the needs of individuals and organizations with the needs of the living ecosystem that shelters them, and to the means of accomplishing such an ambition.

## Figures and Tables

**Table 1:** The Step 2 Change Objective Matrix depicting the DEA-A Functional Profile to achieve for each actor by targeting their Stress-Cognition-Resources-Ecosystem [SCR-E] determinants.

	Feedback			Determinants			
AR Change Objectives		Functionality of AR (+)	Dysfunctionality of AR/ Allostatic Load (−)	At the Internal-Level			At the Ecosystem Level
				S Stress/Homeostatic Imbalances	C Cognitions about Stake/Resources	R Resources at Internal-Level	E Ecosystem Context
BR1.	Actor			BR1s1: BR1s2: …	BR1c1: BR1c2: …	BR1r1: BR1r2: …	BR1e1: BR1e2: …
	Ecosyst.						
CR1.	Actor			CR1s1: CR1s2: …	CR1c1: CR1c2: …	CR1r1: CR1r2: …	CR1e1: CR1e2: …
	Ecosyst.						
BR2.	Actor			BR2s1: BR2s2: …	BR2c1: BR2c2: …	BR2r1: BR2r2: …	BR2e1: BR2e2: …
	Ecosyst.						
ER1.	Actor			ER1s1: ER1s2: …	ER1c1: ER1c2: …	ER1r1: ER1r2: …	ER1e1: ER1e2: …
	Ecosyst.						
…	Actor			…	…	…	…
	Ecosyst.						

AR = Allostatic Regulation; BR = Behavioral Regulation (leading to concrete modifications of the internal/external environment); CR = Cognitive Regulation (leading to reappraise the stake and/or resources); ER = Emotional Regulation (leading to endure/tolerate the arousal); S = Stress (determinants relating to the nature of the homeostatic imbalance(s) which motivate(s) in the sense of a drive both the need for regulation and the direction/expression of regulation, i.e., the force allowing the A/O to be put in movement from its position and instilling in it the need to self-regulate in the direction or in opposite direction to the targeted regulation); C = Cognitions (determinants relating to perception, interpretation or even anticipation at play in the elaboration of both the adaptive stake underpinned by the situation and the control to handle it); R = Resources (determinants bringing together the person’s objective internal resources,—e.g., emotional skills, physical condition, etc.—, to implement/maintain the targeted regulations and resist the pressure of the internal/external environment); E = Ecosystem (determinants relating to the context and the regulations implemented by the other A/O which surround the A/O and configure external resources for it). The colors were used to emphasize the expected functional (green) or dysfunctional (red) nature of the regulation in the A/O situation. The three dots emphasize that as many objectives as necessary should be listed.

**Table 2:** Illustration of techniques that can be mobilized in Step 3 to enable Stress-Cognition-Resources-Ecosystem [SCR-E] changes.

S	C	R	E
— Pharmacological support — Relaxation — Exposure/Desentization — Biofeedback — Cardiac coherence — Mindfulness — Emotion labeling — Emotion acceptance/expression — Paradoxical instructions — …	— Conscious raising/Risk Awareness — Persuasion — Motivational Interviewing — Decisional Balance — Modeling — Role-Play — Cognitive distraction — Framing/reframing — Graded Tasks — Self-Talk — Imaging — Cognitive restructuring — Schema therapy — …	— Education/Increasing knowledge — Enablement — Training/Increasing skills — Monitoring — Problem-Solving — Time management — Body changes (e.g., surgical operation) — Kinesitherapy — Planning/Implementation/Preparation of action — …	— Legislations/regulations — Restriction/limiting environment resources — Guidelines — Restructuring the physical/social environment — Social support/mediation — Avoidance/reducing exposure to cues for the behavior — Adding objects to the environment — Service delivery — Nudge — Financial incentives/aids — …

Note that the table is illustrative (techniques are not exhaustive as depicted by the three dots). Furthermore, only theory-driven techniques are provided. Others derived from the expertise or experience of stakeholders are not provided. S = Stress; C = Cognitions; R = Resources; E = Ecosystem.

## Data Availability

No new data were created or analyzed in this study.

## Supplementary Materials

The following supporting information can be downloaded at: https://www.mdpi.com/article/10.3390/ijerph21030378/s1, File S1: DEA-A Illustrative Vignettes.docx; File S2: Step 1 DEA-A Functional Ecosystem Analysis Grid.pub; File S3: Step 2 DEA-A Matrix of Change Objectives.xlsx.
