# Supplementary material for: Planning Individual and Population-Based Interventions in Global Health: Applying the DEA-A Framework to Promote Behavioral, Emotional, and/or Cognitive Change among Stakeholders
Source: Int J Environ Res Public Health. 2024 Mar 21;21(3):378. doi: 10.3390/ijerph21030378 (PMC10970467; doi:10.3390/ijerph21030378)
Supplement: Supplementary file 1 [file ijerph-21-00378-s001.zip › ijerph-2851680-supplementary/File S1_DEAA Illustrative Vignettes.pdf]

Illustrations of the intervention planning process using a DEA-A framework: vignettes for individual-based (IBi) and population-based interventions (PBi)

The vignettes presented below are intended to illustrate the IBi or PBi planning process based on the DEA-A framework, following the steps and tools presented in Broc et al.' main publication '*Planning individual and population-based interventions with a DEA-A framework: practical guidelines to promote behavioral, emotional and/or cognitive change among stakeholders in a Global Health setting*'.

Guillaume Broc, Jean-Baptiste Fassier, Stéphane Raffard, & Olivier Lareyre

Example of using DEA-A Framework in an IBi context: Integrative management of Obsessive Compulsive Disorder (OCD)[1]

For this example in the context of IBi planning, a patient who met the diagnostic criteria for OCD as a primary diagnosis (Mr. X) is accompanied by a clinical psychologist in an adult psychiatric department. Several introductory sessions were devoted in particular to the development of a therapeutic alliance and the formalization of the patient's request. At this stage, the psychologist will use the DEA-A framework to 1/ establish a functional ecosystem diagnosis of the patient's situation in light of the request thus formulated; 2/ define therapeutic objectives in this context including the patient's environment; 3/ Establish a therapeutic plan with the change techniques envisaged. The clinical interview makes it possible to provide most of the information when it is directed in an evidence-based approach (i.e., the therapist confronting the patient with certain hypotheses based on a triangulation of evidence). Other complementary methods are suggested in the main article.

[BOX S1] Step 1. Logic Model of the Problem – Strategic Committee

The psychologist completes the ecosystem functional analysis grid for his patient (**see Table S1**), by first helping him to specify his request (i.e., distal objective, here very broad with reference to quality of life, but which can be tightened according to the patient's progress in the care process). In the 'ecosystem' section, the therapist identifies the demand/pressures that alter the patient's biopsychosocial homeostasis, at the ontosystem level (e.g., fatigue, cognitive load, fear of separation) as well as other ecosystems (e.g., infantilization, stigma, conflicts at work). He/her also identifies resources at all these levels, either existing (e.g., environment conducive to activities, social and therapeutic support) or lacking to help the patient better adapt (e.g., problems to access psychiatric care infrastructures[2]). The chronosystem part is useful to the practitioner to indicate elements likely to disrupt all or part of the ecosystem, but too recent or imminent to observe the effects at the current follow-up session. In the 'A/O functioning' section, the psychologist 1/ describes the patient's behavioral, cognitive and emotional responses; 2/ infers the positive function of these regulations; 3/ diagnoses their objective impact, both positive and negative, on the patient and their ecosystem[3]. The therapist notes in particular the patient's feeling of vulnerability, the need for recognition, and the great frustration which reflect a significant alteration of his fundamental needs for competence, belonging and autonomy. It also clearly identifies that certain symptoms can be (involuntarily) maintained by the ecosystem, in this case the wife of Mr. X. (e.g., the housekeeping compulsion associated with fear of contamination coincides with Madame X's need to see that household chores are carried out). The same goes for the family accommodation of Madame X to the obsessions of her husband, who comes to carry out his rituals or do things in his place (resulting in further allostatic load for her). Another grid should be filled in for this purpose for the wife of Mr X., requiring an appointment to be made alone or with the patient. The psychologist questions the benefit of also considering the workplace ecosystem as a stakeholder and filling in a dedicated grid. The role of a Strategic Committee or similar is helpful in this type of reflection. The clinician will solicit his/her multidisciplinary staff, or request

[1] The content of this document is only illustrative of the approach and use of the tools described in the original article on the application of the DEA-A framework for intervention planning.

[2] Note that this item could have been positioned as demand/pressure since, from the patient's point of view, this lack would harm the satisfaction of a fundamental need (here, the patient can accommodate difficulties in accessing the psychiatric center, as long as he manages to get an appointment or risk being anxious).

[3] Certain allostatic responses can in this regard have a positive effect on the distal objective and can be reinforced by the program (they do not necessarily appear in this example).

an advisory opinion from a Multidisciplinary Team Meeting including patient representatives (as tends to be deployed via instant messaging).

[BOX S2] Step 2. Program objectives: logic model of change

The psychologist relies on the different functional analysis grids of the ecosystem provided for the case of Mr. X, the information of which he/she cross-references to construct the change matrices for each stakeholder. For example, he/she completes a matrix for his/her patient (**see Table S2**). To do this, the clinician begins by suggesting therapeutic objectives in terms of behavioral, cognitive and/or emotional change ('Allostatic Responses [AR]' in the table). These objectives can be ordered strategically, with one target regulation likely to influence another or having to be prioritized. This is the case here. The psychologist judged, in consultation at least with the patient or even with the staff, that a priority was for Mr. X to maintain the SSRI treatment which he intended to stop against psychiatrist's opinion[4]. Two objectives centered on needs rather than symptoms were then suggested, aiming respectively at the patient's self-affirmation and the planning of restaurant outings with his wife in accordance with the therapist's humanist/ACT approach. With regard to the DEA-A framework, the psychologist expects that the achievement of such objectives will positively reinforce the patient and at the same time prevent him from creating other homeostatic imbalances (by forgetting himself and degrading his relationships as long he gets bogged down in his struggle with OCD). The final objectives are the emotional management of outbursts of anger and, finally, the reduction of the most burdensome rituals. In the event of more severe symptoms, these latter objectives could have been brought forward by the psychologist. The clinician then provides information on the expected impact of these functioning targets, positive and negative, for Mr. X and his ecosystem. These insights are discussed with the patient and support their active involvement in the therapeutic process. They are also possible markers for evaluating the clinical relevance of the intervention. Concerning the allostatic load specific to these target regulations, it is thus prevented as much as possible (e.g., taking into account the adverse effects of SSRIs, the psychologist will report to the psychiatrist who may, if necessary, adapt the dosage or change treatment to Clomipramine). Finally, the psychologist identifies the main SCR-E determinants of the desired change objectives[5]. These will be targeted by the components of the therapeutic intervention. As illustrated in the table, references to other cells of the matrix as well as to the matrices of other stakeholders help to consider the change in its temporal and ecosystem dynamics (e.g., CR1 once reached becomes a resource, hence a determinant of BR2).

[BOX S3] Step 3. Program design

At this stage, the psychologist establishes an intervention plan (i.e., therapeutic plan in clinical practice) which specifies which actor(s) is/are targeted, on what objective(s) of change of their matrix, at what time, but also in what way. The question of 'how' is in fact a central point of step 3. The psychologist plans what will be the components of his intervention with his patient and key stakeholders. To do this, he/she draws on change techniques proven in scientific literature, as well as in his/her experience and that of others (professionals and non-professionals alike). The psychologist decides to involve Mr. X so that he takes ownership of the suggested solutions and is himself a force for creative proposals (this facilitates implementation, in other words the patient's compliance with these strategies). Table 2 of the original article provides some ideas for acting on the targeted SCR-E determinants of each change objective. Consider for example how to increase the probability of achieving objective BR3 (i.e., reduce the most burdensome rituals). The psychologist plans to use Mindfulness to promote BR3s1 and BR3s2 by helping the patient identify their feelings and bodily sensations, then Biofeedback to strengthen distress tolerance. In a second strop, He/she favors exposure therapy (e.g., by confronting the patient his thoughts, images that make him anxious and

[4] Even if the psychologist agrees and believes that CBT treatment is superior to SSRIs, he recommends that the patient meet the psychiatrist before any decision is made.

[5] To facilitate the identification of the determinants, it may be helpful to refer to Table 1 of the conceptual article on the DEA-A framework (Broc, Brunel & Lareyre in this special issue).

provoke his obsessions without ritualizing) to support BR3c1 and BR3c2, and reeducation to respond to BR3r1. The psychologist finally suggests modifications to be made in the environment for BR3e1, such as replacing the lever handles with doorknobs to bypass the verification OCDs. BR3e2 has already been addressed by a specific intervention targeting the wife of Mr. X. With the overview offered by the matrices, the psychologist can identify which change techniques advantageously make it possible to simultaneously target several SCR-E determinants and/or several change objectives.

Table S1. Step 1 Functional analysis grid of adaptation according to a DEA-A framework in context of OCD integrative care.

Functional analysis grid of an actor/organization (A/O)

A/O's name (if applicable):	Mr. X,	Distal Goal:	Enabling quality of life with OCD
Category :	Patient	Date / Follow-up:	20/12/23 / Sixth session

Ecosystem relevant characteristics

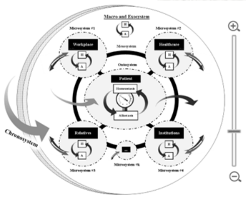

At the system-level:		Demands	Resources
**Onto**	A/O Internal-level	Increased obsessions and compulsions; Mental suffering; Fatigue; Cognitive difficulties; Digestive disorders; Loss of sexual desire; Fear of being abandoned by his wife;	Significant effects of taking SSRIs on depressive episodes linked to OCD (although he is no longer using them and suffers from digestive side effects); Lives in a lively neighborhood conducive to activities; Willingness to get by and enjoy life with his spouse; Mobility issues in accessing psychiatric care infrastructures.
**Micro**	Relationship <u>with</u> others A/O	Partner placed additional pressure during certain compulsions (e.g., becoming impatient, doing instead of, or forcing to); Infantilization; Conflict with management and colleagues	He and his wife share a longtime friend and confidant ; Support from the general practitioner for the recognition of OCD as a long-term condition (financial coverage of care).
**Meso**	Relationship <u>between</u> others A/O	/	Facilitating communication between the psychiatrist, the clinical psychologist and the family; Lack of communication between occupational medicine and psychotherapists
**Exo**	Political/Institutional context	Complicated appointments at the adult psychiatric service proving to be a real source of anxiety for the patient	/
**Macro**	Societal/cultural context	Stigmatization of people living with OCD (mainly teasing and social distancing from people in his environment, , which limits his activities outside)	/
**Chrono**	Transitions/Evolutions	/	New social tendency to affirm and better accept mental disorders and neuroatypicalities; A friend has just moved to the area ; An integrated health and social services center has just opened near his home

A/O Functioning profile

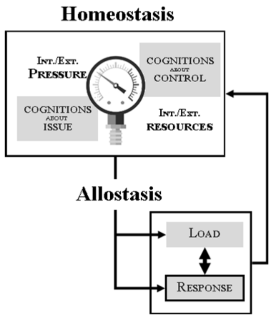

Allostatic regulations		Underlying stress /Homeostasis imbalances	Positive impact		Negative impact/Allost. load	
			On the A/O itself	On its ecosystem	On the A/O itself	On its ecosystem
**Behav.**	Compulsions (operating the handle in sequences of three, ritualized hand washing)	Fear of being responsible for a disaster / Getting sick	Temporarily relieves obsessions	Active participation in household chores	Fatigue ; Waste of time ; Gets often late on schedules	Relatives especially his wife get annoyed; Family accommodation ; Gets co-workers in trouble ; Direction is overwhelmed / cannot handle crises
**Cog.**	Obsessions (dirt and contamination, being responsible for the death of a loved one) ; Cognitive Withdrawal	Feeling of helplessness/ insecurity	Fuels sense of control over environmental contingencies	/	Cognitive overload ; Panic attacks ; Concentration issues	Worries family and relatives ; Friends don't feel listened to/ considered
**Emot.**	Emotional substitution (tantrums against his wife)	Frustration; Feeling of not being understood/accepted	Way to call for help / to seek recognition	/	Feeling guilty	Argument with his wife ; Wife feel sadness and helplessness

Table S2. The Step 2 Change Objective DEA-A Matrix for a patient in context of OCD integrative care.

		Feedback		Determinants			
				at the internal-level			at the Ecosystem level
**AR change objectives**		**Functionality of AR (+)**	**Dysfunctionality of AR / Allostatic load (−)**	**S Stress/homeostatic imbalances**	**C Cognitions about stake/resources**	**R Resources at internal-level**	**E Ecosystem context**
BR1. The Patient (P) maintains SSRI antidepressant intake awaiting medical advice	***Actor***	Regulation of depressed mood/Positive thoughts and increased energy	Digestive disorders (nausea and constipation) / Begins to limit activity (outings, intimate life)	BR1s1. P is satisfied with the progress made since pharmacological treatment<br><br>BR1s2. P fears relapsing without medication<br><br>BR1s3. P fears making a decision without medical advice	BR1c1. P is aware of the benefits of pharmacological treatment<br><br>BR1c2. P considers that the absence of symptoms could be linked to the treatment and not to remission	BR1r1. P has sufficient persistence capacity to maintain drug intake<br><br>BR1r2. P knows the etiology of OCD and how it works	BR1e1. The psychologist, with P or his agreement, contacts the psychiatrist regarding antidepressant treatment (see Psychologist's Matrix BR1)
	Ecosyst.	More pleasant relationship with P on a daily basis					
CR1. P is more assertive (self-affirmation)	***Actor***	Actor of his needs and care/ less frustration		CR1s1. P needs to feel autonomous<br><br>CR1s2. P needs to get back into a healthy husband/wife relationship<br><br>CR1s3. P is not ashamed of having difficulties	CR1c2. P has confidence in his abilities to do things on his own<br><br>CR1c3. P does not blame himself for having OCD (acceptance)	CR1r1. P has the ability to tolerate the social gaze<br><br>CR1r2. P better identifies his needs	CR1e1. P's wife no longer does things for him (see Wife's Matrix CR2 concerning family accommodation of symptoms)<br><br>CR1e2. The mutual friend help with mediation in the couple
	Ecosyst.	Active involvement in therapeutic work with the psychologist	Likely problematic upheaval of each person's place in the marital relationship				
BR2. P regularly goes out to restaurants with his wife	***Actor***	Renewing ties between spouses; satisfy one's needs/take care of themselves/ Exposure therapy	Costly in resources and energy / can worsen the condition if negative experience (requires attentive psychological support)	BR2s1. P wants to spend quality time with his wife and reconnect with her	BR2c1. P has a strong self-efficacy perception of carrying out the activity	BR2r1. P is able to assert himself (cf. CR1)<br><br>BR2r2. P has sufficient inhibition capacities	BR2e1. There are more discreet restaurants to start exposure gradually<br><br>BR2e2. Making an appointment at the psychiatry department is made easier<br><br>BR2e3. Solutions are provided in terms of transport/mobility to access care
	Ecosyst.	Renewing ties between spouses/ Wife satisfies her needs/takes care of herself					
ER1. P manages his outbursts of anger	***Actor***	Avoid feeling guilty/greater consideration from others		ER1s1. P feels confused when he loses his temper<br><br>ER1s2. P feels pride when he handles the situation without conflict	ER1c1. P perceives the disproportionate nature of certain reactions<br><br>ER1c2. P is convinced	ER1r1. P has efficient interactional resources (empathy, communication skills)	ER1e1. P's wife maintains an open posture (see Wife's Matrix CR3)
	Ecosyst.	More pleasant relationship with P on a daily basis					

					by the negative effect his anger has on his personal, social and professional life	ER1r2. P has skills in emotional self-regulation	
BR3. P no longer perform the most burdensome rituals (e.g., the compulsion to activate the doorknob)	***Actor***	Improved personal, social and professional quality of life	Panic attacks may occur at first	BR3s1. P feels uncomfortable practicing such rituals<br><br>BR3s2. P tolerates stress resulting from not performing rituals	BR3c1. P realizes that he has no control over the misfortunes that happen to his relatives<br><br>BR3c2. P identifies his cognitive schemas linked to OCD (e.g., false beliefs he has developed about himself and the outside world)<br><br>BR3c3. P perceives the usefulness and feasibility for him to do so	BR3r1. P has proficient executive functions (e.g. inhibition capacities)	BR3e1. P is exposed/confronted with an unusual environment<br><br>BR3e2. P's wife maintains an open posture and do not interact (see Wife's Matrix CR2 and CR3)
	Ecosyst.	Reduces sources of tension between spouses / Improves the working conditions of colleagues/managers in the company where P works					

***BR**=Behavioral Regulation (leading to concrete modifications of the internal/external environment); **CR**=Cognitive Regulation (leading to reappraise the stake and/or resources); **ER**=Emotional Regulation (leading to endure/tolerate the arousal); **S**=Stress (determinants relating to the nature of the homeostatic imbalance(s) which motivate(s) in the sense of a drive both the need for regulation and the direction/expression of regulation, i.e., the force allowing the A/O to be put in movement from its position and instilling in it the need to self-regulate in the direction or in opposite direction to the targeted regulation); **C**=Cognitions (determinants relating to perception, interpretation or even anticipation at play in the elaboration of both the adaptive stake underpinned by the situation and the control to handle it); **R**=Resources (determinants bringing together the person's objective internal resources, –e.g., emotional skills, physical condition, etc.–, to implement/maintain the targeted regulations and resist the pressure of the internal/external environment); **E**=Ecosystem (determinants relating to the context and the regulations implemented by the other A/O which surround the A/O and configure external resources for it).*

Example of Using DEA-A Framework in a PBi context: Development of a strategy for optimizing the diagnostic pathway in Endometriosis[6]

This example completes the previous illustration in an IBi context, this time if in a population intervention. A Project Manager in a Public Health Authority's mission is to propose a program aimed at optimizing the diagnostic pathway for women living with endometriosis. He/she will use the method and tools of the DEA-A framework to 1/ establish a functional ecosystem diagnosis of the problem with regard to the distal objective; 2/ define the change objectives for each category of stakeholder; 3/ design the program.

[BOX S4] Step 1. Logic Model of the Problem – Strategic Committee.

The Project Manager first identifies who the stakeholders will be (e.g., patients, general practitioners [GPs], gynecologists, medical advisors). He/She sets up a Strategic Committee where these categories of stakeholders will be represented and will give (in particular) an advisory opinion at each stage of planning. The Project Manager then undertakes a functional and ecosystem analysis of the situation linked to diagnostic wandering in Endometriosis. He/She fills in a grid taken from the DEA-A framework for each category of stakeholders (an example is given for GPs in **Table S3**). The information it provides can be drawn from the synthesis of literature research, qualitative interviews (individuals and focus groups) and/or other methods described in the original article. If we take the 'GP' grid, the Project Manager first recalls the distal objective, which will later be broken down into change objectives (see step 2). Then, he/she identifies in the "ecosystem" section the demands that challenge the homeostasis of GPs within and outside their own ontosystem. The Project Manager is particularly interested in those which affect the GPs' fundamental needs of competence (e.g., feeling of being overwhelmed), relatedness (e.g., disconnection with patients, coordination issues with other caregivers) and autonomy (e.g., pressure from insurance companies on GPs)[7]. He/She also identifies resources at all levels of the GP ecosystem, either existing (e.g. guidance made by expert centers; facilitating doctor/patient relationship) or missing which would allow them to better function (e.g. lack of interaction with pharmacists[8]). The chronosystem part is particularly suitable for representing the elements of instability (imminent or ongoing change) in the ecosystem. The Project Manager notes for example in this section the constant evolution of the diagnostic offer likely to change the facts. In the "A/O Functioning" section, the Project Manager 1/ delineates the behavioral, cognitive, and emotional responses of GPs; 2/ infers the positive function of these regulations; 3/ diagnoses their objective impact, both positive and negative, on GPs and their ecosystem. The Project Manager raises fundamental violations of GPs' needs for autonomy (e.g., pressure to be accountable), competence (e.g., feeling of helplessness) and belonging (e.g., questions about their vocation, their relationship

[6] The content of this document is only illustrative of the approach and use of the tools described in the original article on the application of the DEA-A framework for intervention planning.

[7] The actor's status in a PBi context is taken as a reference. Thus, as a physician, a GP may feel his/her fundamental need for autonomy threatened (e.g., when having to justify his/her sick leave prescriptions), without the same being true in his/her private life (where he/she cannot be accountable to no one). The compartmentalization of identity facets is shown to be protective in that sense. All considered, these singularities are smoothed out in population-based approaches which are above all nomothetic.

[8] Note that this item could have been positioned as demand/pressure since, from the GPs' viewpoint, the absence of such a resource was detrimental to their homeostatic balance, e.g., if it were to undermine their efforts, hence limit their agency on the question of diagnosis endometriosis (i.e., violation of the fundamental need for competence). In other words, contact with pharmacists should be considered as a factor of plasticity (plus-value) rather than stability (sine qua none condition), at least for GPs (this may not be true for another actor).

with patients), to which the latter adapt in different ways (see 'allostatic regulations' column). These adaptations have a weight on the homeostasis of the actors, and ultimately on the diagnostic process[9]. It can be noted that the same regulation (e.g., hastening medical questioning and auscultation) can be double-edged for GPs (satisfaction at having honored all consultations, but frustration with the quality of the care provided), but also benefit a actor (e.g., secretary/patients in waiting room) and harm another (woman awaiting diagnosis) for these same reasons. Certain allostasis can thus be maintained because they suits with part of the ecosystem. At this stage, the Project Manager consults the group of stakeholders to consider whether a functional analysis should be undertaken for other categories of actors having emerged from the findings (e.g., pharmacists, consultation secretaries), and whether they must be approached to join the Strategic Committee.

[BOX S5] Step 2. Program objectives: logic model of change

A change matrix is completed for each category of stakeholder based on all the ecosystem functional analysis grids provided. Concerning for example the GPs matrix (see Table S4), the Project Manager first establishes in row a list of behavioral, cognitive and/or emotional change objectives ("Allostatic Responses [AR]" in the table), the achievement of which will promote access to the diagnosis in the context of Endometriosis[10]. He/She considers them in a processual logic/sequence, e.g., ER1 (prevention of inappropriate emotion responses) helping to determine CR1 (open posture) then, both, BR1 (giving more space to the patient and his request at the time of the medical questioning, while managing the stress caused by possible delays in consultations). These links may be mentioned like here by references to other cells of the matrix as well as to other actor matrices. The other interest envisaged by the PH is to ensure as a priority the patient's psychological care (ER1, CR1 and BR1) before any medical diagnosis process (BR1, BR2, BR3). Such a process can be lengthy, challenging, and uncertain, hence the need to compensate or prevent certain homeostatic imbalances in patients. In a first column section, the PH anticipates the positive and negative impact of each target change, both for GPs and their ecosystem. He/She is thus better prepared for possible difficulties in implantation, as well as for potential adverse effects of the program linked to allostatic loads. Some risks can even be attempted to be reduced. For example, the threat of a disruption of care, shared by CR1 and BR3, could be prevented by involving consultation secretaries (a dedicated matrix is precisely planned), or even political stakeholders (i.e., those best placed to intervene at the level of exo, macro and chronosystem in PBi contexts). Finally, the Project Manager identifies the main SCR-E determinants of the desired change objectives (still from the grids of step 1) that will be further targeted by the program components.

[BOX S6] Step 3. Program design

The Project Manager determines an intervention plan, which describes in particular which active principles of the program will act at what time on this or that change objectives of the identified stakeholders. The DEA-A matrices provided here are already indicative of the process by which the different targeted allostasis are likely to occur within the ecosystem (e.g., by listing in sequence the objectives of change, the SCR-E determinants of these operations, the possible junctions within and between the actor matrices, etc.). The Project Manager selects from among the change techniques (see e.g. Table 2 of the original article) the most appropriate ones, which would ideally target several SCR-E determinants and/or several change objectives at the same time. Let's consider the ER1 objective (i.e., remain calm in front of the patient) for an illustration. For ER1c1 and ER1s1, the Project Manager plans supervised role-playing games with modeling allowing GPs to experience different situations. He/She will rely on conscious-raising sessions for ER1c2, and on the training of GPs in

---

[9] Certain allostatic responses can in this regard have a positive effect on the distal objective and can be reinforced by the program (they do not necessarily appear in this example).

[10] Given the heterogeneity in the functioning of the actors, it is more than likely that certain GPs have already adopted one or the other, or even all of these target regulations. Others, however, are very far from it, hence the interest of the DEA-A matrix in discriminating constrained parameters (i.e., target allostatic responses) from free parameters (unspecified, i.e., degree of freedom left to stakeholders in their way of functioning).

patient-centered approaches for ER1r1, ER1r2 and ER1r3. Concerning ER1e1, the objective is already addressed in the Secretariat's Matrix, particularly in terms of resources (supervision of organizational aspects, provision of suitable equipment/software). On the allostatic load (disruption of care), the Project Manager plans, among other things, environmental restructuring of the medical office (e.g., light openings, aquatic pool, vegetation, background music) to help both patients and staff to accommodate with delays. It will then remain for the Project Manager to articulate everything into a program, and to address the remaining phases of planning (i.e., Program production, Implementation Plan, Evaluation Plan).

Table S3 (a). Step 1 Functional analysis grid of adaptation according to a DEA-A framework in context of Endometriosis Diagnostic Wandering.

Functional analysis grid of an actor/organization (A/O)

A/O's name (if applicable):	/	Distal Goal:	Optimizing the diagnostic pathway / Decreasing diagnostic wandering in Endometriosis
Category :	General practitioner	Date / Follow-up:	20/12/23

Ecosystem relevant characteristics

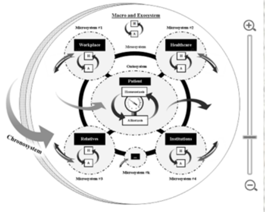

At the system-level:		Demands	Resources
**Onto**	A/O Internal-level	Workload ; Overlap between work and non-work life; Feeling helpless and overwhelmed (lack of knowledge about diagnostic procedures); Psychological exhaustion/burnout; Perception of hindered work	Personal, family and social life (when preserved); Flexibility; Resilience/emotional management skills; Ability to work under pressure (habit); Facilitating administrative secretariat
**Micro**	Relationship <u>with</u> others A/O	Relationship with patients (multiple consultations, reprimands, emotional contagion, high or even unrealistic expectations, distrust)	A facilitating doctor/patient relationship; Quality of communication with gynecologists and expert radiologists (for the referral of patients); Direct communication between caregivers via instant messaging application (for certain GPs); Lack of interaction with some primary-care providers such as pharmacists.
**Meso**	Relationship <u>between</u> others A/O	Indirect pressure from certain mutuals or insurance companies from patients claiming for their rights (certificate signature, completeness of files, commitment to liability, etc.)	Guidance made by expert centers/patient associations; Quality of patient monitoring by other health professionals
**Exo**	Political/Institutional context	Legislation (e.g., law implying the territorial responsibility of private doctors to provide duty in public hospitals); Tightened conditions for prescribing sick leave or covering certain medical examinations; Difficulties in patients' access to care (shortage of caregivers, medical desertification, precariousness); Deterioration of GPs' working conditions.	National guidelines ; National priority/Territorial strategies (Endometriosis Plan)
**Macro**	Societal/cultural context	Predominance of social networks (fads, over-media coverage); Judicialization of medical error (no right to make mistakes, hyper-responsibility); Preference for non-conventional medicines; Normative trends towards self-medication; Clientelism in care	Social media: greater recognition of Endometriosis (change in mentalities); Progressivism (suffering and needs of women better considered)
**Chrono**	Transitions/Evolutions	COVID-19 pandemic; Deployment of medical teleconsultation	Constant progress in the diagnostic offer for endometriosis

Table S3 (b). Step 1 Functional analysis grid of adaptation according to a DEA-A framework in context of Endometriosis Diagnostic Wandering.

A/O Functioning profile

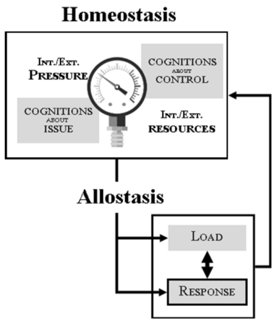

Allostatic regulations		Underlying stress /Homeostasis imbalances	Positive impact		Negative impact/Allost. load	
			On the A/O itself	On its ecosystem	On the A/O itself	On its ecosystem
**Behav.**	Treatment of symptoms rather than investigating an underlying chronic pathology ;<br><br>Referral to specialists may be inadequate, leading to unnecessary examinations and increased patient costs;<br><br>Care coordination shows heterogeneity (with some GPs providing follow-up only at the patient's initiative or discontinuing investigations);<br><br>Some GPs botch the medical questioning of the patient or even auscultation.	Cognitive load; Fatigue/ Psychological exhaustion; Feeling of helplessness; Weariness ; Doubt and questioning	Protects against negative feedback; Regulation of the workload ; Impression of having provided a solution to the complaint	The medical office is likely to fulfill today's consultations<br><br>Reduction of time spent in the waiting room for patients	Perceive himself to be hindered in his work	Diagnostic wandering / Delay in care affecting the patient and her family, social and professional ecosystem; Avoidable increase in public health spending; Other specialists potentially put in difficulty
**Cog.**	Trivialization of patient complaints Psychiatrization of endometriosis ; Form of rigidity (do not deviate from the guidelines)	Anxiety of following patients with serious illnesses ; Guilt about delivering bad news; Weariness	Passive avoidance of anxiety-provoking situations; Normalization of the situation; De-responsibility	Reassures both the patient and relatives in the early stages	/	Patient may blame themselves / not feel listened to; Worse prognosis for Endometriosis Induces/Increases resistance in the doctor-patient relationship
**Emot.**	Apathetic withdrawal / Indifference ; Passive aggression (anger, irritation, annoyance) towards certain patients	Emotional distress towards the patient (sadness, feeling of injustice, embarrassment); Fatigue/ Psychological exhaustion; Frustrations/ Disappointments; Guilt/ Regrets ; Reactance (doesn't like being told how to do his job)	Emotional discharge; Psychological protection (burnout prevention)	Limitation of emotional contagion for GP's Family and relatives	Guilt when limits are exceeded	Patient may not feel recognized/ understood ; Induces/Increases resistance in the doctor-patient relationship

Table S4. The Step 2 Change Objective DEA-A Matrix for General Practitioner in context of Endometriosis Diagnostic Wandering

		Feedback		Determinants			
				at the internal-level			at the Ecosystem level
**AR change objectives**		**Functionality of AR (+)**	**Dysfunctionality of AR / Allostatic load (–)**	**S Stress/homeostatic imbalances**	**C Cognitions about stake/resources**	**R Resources at internal-level**	**E Ecosystem context**
ER1. GP prevents inappropriate emotional responses (apathetic, antipathetic) towards his patient in consultation	***Actor***		Emotional contagion / overload; Psychological exhaustion	ER1s1. GP manages Professional stress<br><br>ER1s2. GP feels confused when he loses his temper<br><br>ER1s3. GP expresses his/her emotions	ER1c1. GP does not perceive himself to be hindered in his work<br><br>ER1c2. GP perceives the meaning of his work<br><br>ER1c3. GP is aware and accepts his/her limits in making diagnosis	ER1r1. GP has skills in emotional expression<br><br>ER1r2. GP has skills in emotional acceptance/management.	ER1e1. The GP environment allows emotional relief<br><br>ER1e2. Non-medical workload (e.g., administrative burden) is handled or postpone (<u>see Secretariat's Matrix BR2</u>)<br><br>ER1e3. The patient has realistic expectations of care (<u>see Patient's Matrix CR3</u>)
	Ecosyst.	Feeling of being recognized/listened to					
CR1. GP adopts an open posture (does not psychiatrize or trivialize the complaint)	***Actor***	Therapeutic Alliance / Patient's confidences on key symptoms	Extension of consultation time	CR1s1. GP feels pride in keeping humanized the doctor-patient relationship	CR1c1. GP sees the benefit/the meaning of listening to the patient<br><br>CR1c2. GP is aware that non-specific symptoms (digestive disorders, fatigue, pain) sometimes hide real pathologies	CR1r1. GP has empathetic and reflective listening skills (cf. <u>ER1</u>)<br><br>CR1r2. GP knows the determinants of the therapeutic alliance<br><br>CR1r3. GP knows how to handle resistance in communication	CR1e1.The secretariat plans consultations (durations, frequencies) in a manner adapted to medical follow-up (<u>see Secretariat's Matrix BR1</u>)
	Ecosyst.	Patient's feeling of being recognized	Disruption of care				
BR1. GP allots the necessary time for the medical questioning and examination of the patient.	***Actor***	Incidental diagnosis		BR1s1. GP feels embarrassed when he doesn't examine a patient adequately	BR1c1. GP identifies medical questioning and auscultation as a medical routine and priority	BR1r1. GP is able to allocate the necessary time<br><br>BR1r2. GP has required self-determination capabilities	BR1e1. Non-medical workload (e.g., administrative burden) is handled or postpone (<u>see Secretariat's Matrix BR2</u>)
	Ecosyst.	Patient's Feeling of being considered / Incidental diagnosis					
BR2. GP ensures coordination of the diagnostic investigation when referring to other specialists	***Actor***	Always has the most recent information to progress in diagnosis	Additional cognitive load	BR2s1. GP experiences satisfaction when solutions are found for his/her patients' issues.<br><br>BR2s2. GP is curious and interested in	BR2c1. GP believes that it can thus facilitate access to a diagnosis	BR2r1. GP has the necessary tools to ensure the coordination<br><br>BR2r2. GP has	BR2e1. The secretariat assists the GP in planning follow-up appointments (<u>see Secretariat's Matrix</u>

	Ecosyst.	The patient is relieved of this role/Feels supported; Centralization of the diagnostic process for specialists		the outcome.<br><br>BR2s3. GP enjoys feeling useful and finding meaning in his/her vocation	BR2c2.GP is convinced of the legitimacy of the complaint / believes the patient	monitoring skills	BR1)
BR3. GP orders an MRI at the Expert Center	***Actor***	Confirm/Refute the diagnosis		BR3s1. GP feels a desire to do well for his patient	BR3c1. GP is convinced that the MRI reading must be done by an Endometriosis specialist<br><br>BR3c2. GP is confident in the benefit of an MRI	BR3r1. GP knows an Endometriosis Expert Center<br><br>BR3r2. GP has technical knowledge about Endometriosis	BR3e1. There is an Endometriosis Expert Center nearby<br><br>BR3e1. There are specialists in MRI reading for endometriosis in the Region
	Ecosyst.	Patient's Feeling of being considered / Confirm/Refute the diagnosis	Cost for Health Insurance system / Increased wait time in radiology departments (disruption of care)				

****BR**=Behavioral Regulation (leading to concrete modifications of the internal/external environment); **CR**=Cognitive Regulation (leading to reappraise the stake and/or resources); **ER**=Emotional Regulation (leading to endure/tolerate the arousal); **S**=Stress (determinants relating to the nature of the homeostatic imbalance(s) which motivate(s) in the sense of a drive both the need for regulation and the direction/expression of regulation, i.e., the force allowing the A/O to be put in movement from its position and instilling in it the need to self-regulate in the direction or in opposite direction to the targeted regulation); **C**=Cognitions (determinants relating to perception, interpretation or even anticipation at play in the elaboration of both the adaptive stake underpinned by the situation and the control to handle it); **R**=Resources (determinants bringing together the person's objective internal resources, –e.g., emotional skills, physical condition, etc.–, to implement/maintain the targeted regulations and resist the pressure of the internal/external environment); **E**=Ecosystem (determinants relating to the context and the regulations implemented by the other A/O which surround the A/O and configure external resources for it).*
